# Real-time dosimetry with radiochromic films

**DOI:** 10.1038/s41598-019-41705-0

**Published:** 2019-03-29

**Authors:** Pierluigi Casolaro, Luigi Campajola, Giovanni Breglio, Salvatore Buontempo, Marco Consales, Andrea Cusano, Antonello Cutolo, Francesco Di Capua, Francesco Fienga, Patrizio Vaiano

**Affiliations:** 1 University of Napoli Federico II, Department of Physics, I-80126 Napoli, Italy; 2 Istituto Nazionale di Fisica Nucleare (INFN) - Sezione di Napoli, I-80126 Napoli, Italy; 3 University of Napoli Federico II, Department of Electronical Engineering, I-80125 Napoli, Italy; 4 Optoelectronics Group - Department of Engineering, University of Sannio, I-82100 Benevento, Italy

## Abstract

Radiochromic film dosimetry has been widely employed in most of the applications of radiation physics for over twenty years. This is due to a number of appealing features of radiochromic films, such as reliability, accuracy, ease of use and cost. However, current radiochromic film reading techniques, based on the use of commercial densitometers and scanners, provide values of dose only after the exposure of the films to radiation. In this work, an innovative methodology for the real-time reading of radiochromic films is proposed for some specific applications. The new methodology is based on opto-electronic instrumentation that makes use of an optical fiber probe for the determination of optical changes of the films induced by radiation and allows measurements of dose with high degree of precision and accuracy. Furthermore, it has been demonstrated that the dynamic range of some kinds of films, such as the EBT3 Gafchromic films (intensively used in medical physics), can be extended by more than one order of magnitude. Owing to the numerous advantages with respect to the commonly used reading techniques, a National Patent was filed in January 2018.

## Introduction

Radiochromic films (RCFs) are dosimeters based on the property of modifying the structural characteristics of their crystalline sensitive element when exposed to ionizing radiations. The interaction of ionizing radiation with the film produces a polymerization process in the monomers of the sensitive element^1^. This microscopic phenomenon is reflected in a color change at macroscopic level and the color of the film can be related to the radiation dose. The first work on the employment of solid solutions showing radio-synthesis after the exposure to radiations dates back to 1965 by Mc Laughing *et al*.^2^. The subsequent works on RCFs can be attributed to the National Institute of Standard and Technology (NIST)^1,3,4^. The first prototypes of RCFs were sensitive in the range of dose from a few to hundreds of kGy and therefore these films were employed for high-dose applications only. A kind of radiochromic medium sensitive to lower doses (up to 5 Gy), known as Gafchromic film, was developed in 1986 by the International Speciality Products Inc. (ISP)^1^.

Nowadays RCF dosimetry is considered as a reliable technique for accurate dose assessment and quality checks in many applications of radiation physics. Several types of films are currently available on the market, covering a very wide range of dose from a few mGy to hundreds of kGy^5^. The main application is in medical physics, although RCFs are commonly used also in other fields, e.g. studies of radiation damage of electronic devices, beam diagnostics, radiation processing and radiation-induced sterilization (food and medical instrumentation). Such a wide use is consequence of the intrinsic characteristics of the films. Today RCFs are reliable, easy to use, cheap, portable and non-invasive instruments, providing accurate and permanent values of dose^6^. It has to be noticed that this last feature is very important for legal aspects related to dosimetry. RCFs have very high spatial resolution and moreover most of RCFs types are made of tissue-equivalent materials. These characteristics fit perfectly for the medical physics, in particular for the determination of percentage depth-dose curves in radiotherapy with photon and ion beams^7–11^. RCFs are sensitive to all kinds of ionizing radiation employed in the applications (photons, electrons, protons and heavy ions). The response of the films is almost completely independent of dose-rate, while the energy dependence is more complicated, although it was studied in depth in several works reported in literature. More details on the energy dependence of RCFs are given in the next section.

Despite such appealing characteristics, RCFs cannot be employed as dosimeters or detectors if real-time information are needed. The current RCFs reading techniques, mainly based on commercial densitometers, scanners and spectrophotometers^12–14^, provide dose measurements only after the exposure of the films to radiation. In the practice, the reading is carried out after the withdrawal of the film from the irradiation site. Therefore, the current reading techniques don’t allow measurements of the trend of dose in time, but only integrated dose values. In the applications this important limitation is overcome (when needed) by using other detectors providing the response in real-time. As an example, gas-filled chambers, scintillation detectors, phosphor screens and semiconductor detectors are usually employed for this purpose; however, RCFs may be used in combination with these detectors. The most widespread reading technique based on flat-bed scanners is affected by reproducibility issues, including the warm-up, uniformity and resolution of the scanner, as well as the dependence on the film size, orientation and region of interest for the analysis, lateral non-uniformity and polarization effect. All these aspects have been widely discussed in literature, leading to uncertainties that normally are around 3–6%^15–17^.

In this work, an innovative method, based on opto-electronic instrumentation, is proposed for the real-time reading of RCFs. Previous works on this topic are reported by Mignani *et al*. and Rink *et al*.^18,19^. together with a U.S. patent^20^. The method proposed here has several new interesting features with respect to the existing RCF reading techniques. Because it makes direct use of commercial RCFs (cut in small pieces), it avoids carrying out complicated operations of deposition of radiochormic media on the tip of the fiber. Moreover, with respect to the above-quoted RCF real-time techniques, based on single-use probes, this method uses an optical fiber probe, in which the RCF is disposable and can be quickly replaced with other RCFs in order to make other measurements. This allows the preservation of all the above-discussed characteristics of the films. The advantages of using an optical-fiber-based instrumentation instead of commercial RCF reading tools, not only allow the performance of the reading of the dose in real-time, but also the exploitation of most of the potential of the RCF dosimeters. In fact, as will be demonstrated in this work, measurements of dose can be performed with a very high degree of precision and accuracy. Furthermore, the range of sensitivity of some types of films can be extended by several orders of magnitude, with respect to that declared by the manufacturer. The originality, the technological advances with respect to the existing RCF reading methods and the potential distribution on the market made the invention patentable. For these reason a National Patent was filed in January 2018.

In the next section an overview of the main characteristics of RCFs will be discussed: the operating principle and the response of the films to radiation type, energy and dose-rate. Moreover, different types of films will be shown in relation to their specific applications. Following this, the principle and the experimental set-up of the innovative RCF reading method will be shown and discussed. Finally, in the last section, the operation of the invented method will be demonstrated for two types of films widely employed in the applications: EBT3 and XR-QA2 Gafchromic films exposed to ^60^Co gamma rays at two dose-rates.

### Radiochromic films

This section gives an overview of the main characteristics and application fields of the RCFs. The operating principle and the dependences on relevant physical quantities such as radiation type, energy and dose-rate are briefly described, while more details can be found in the referenced publications. The characteristics discussed here have made RCF dosimetry such a widely spread dosimetry technique in several application fields. Therefore, the proposed RCF real-time reading method adds a significant value to the features of this dosimeter.

### Operating principle and radiation type, energy and dose-rate dependence

RCFs consist of a single or double layer of radiation-sensitive material on a thin polyester base with a coating. The last could be transparent or opaque^5^. The ionizing radiation produces a polymerization process in the monomers of the active layer, resulting in a modification of the optical properties of the film. The materials responsible for RCFs coloration are the dyacetilenes, particular type of crystalline polyacetylenes that show excellent sensitivity to ionizing radiation. Differently from silver halide films, RCFs are self-developing, namely they don’t need chemical or physical processing after irradiation^9,15^.

RCFs are sensitive to almost any type of ionizing radiation (photons, electrons and charged particles over a wide range of energy and dose), being at the same time almost totally insensitive to room light. The response of most of RCFs shows very low dependence on the radiation quality, at least for low-LET (Linear Energy Transfer) radiation (e.g. MeV-energy electrons and photons) usually employed in radiotherapy and radiation hardness applications^21–23^. RCFs energy dependence to high-LET radiation is more complicated. Nevertheless the response of the films to different LET-components of charged particle beams can be taken into account by applying relative effectiveness factors. For example, some types of RCFs, such as EBT2 and EBT3 Gafchromic films, intensively used in radiotherapy with photon, electron and also ion beams, are known for underestimate the dose in the Bragg Peak region. This is known in literature as “quenching effect”. However, RCFs are successfully and commonly employed for the determination of percentage depth dose profiles of photon and ion beam in radiotherapy^24–28^. Concerning RCFs dose-rate dependence, no difference in response has been observed. The sensitivity to neutrons is very low: to date results about RCFs neutron sensitivity are reported by Bazioglou *et al*.^29^. In the practice, RCFs are not used for measuring neutron dose and neutron beam profiles as it is usually done for the other radiations.

### Types of films and related applications

**Figure 1:**
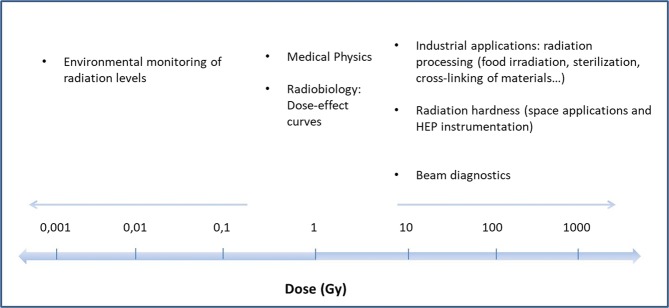
Applications of the radiation physics in which RCFs are employed.

The types of RCFs available on the market cover a wide range of dose, from a few mGy to several hundreds of kGy. As a consequence of this and of the above-mentioned characteristics in comparison to other dosimeters and detectors, RCFs are currently employed in many applications of radiation physics. Figure 1 shows the application fields in which RCFs are use as a function of the dose in Gy. All dose values reported in this work are referred to water, that is the standard for the medical physics. RCFs are used in the dose range from a few tens to hundreds of mGy for quality checks of typical diagnostic instrumentation such as X-ray tube for radiography and Computed Tomography (CT). For these purposes, the ISP provides the Gafchromic series XR. In particular, XR-QA2 films are suited for radiology quality assurance tests, XR-CT2 measure beam slice width in CT scanner, XR-M2 films are dedicated to mammography tests and XR-RV3 are used for peak skin dose measurement^30^. These films are made of an active layer of about 25 *μ*m between a layer of yellow polyester of 97 *μ*m and a layer of white polyester (opaque layer). It has to be noticed that the structure of the films is an important parameter for the real-time reading method, as will be explained in the following section. RCFs are used in the range from hundreds of mGy to tens of Gy for measuring doses typical of external beam therapy with photon and ion beams. The film dosimetry has been adopted as successful method by several hospitals, clinics and centres for cancer therapy^31^. The brachytherapy application deals with doses up to hundreds of Gy and RCFs are employed for dose verification also in this field^32–34^. EBT3 Gafchromic films are intensively used in the medical physics for the determination of depth-dose curves of photon, electron and ion beams. These films are made of an active layer of about 28 *μ*m sandwiched between two polyester layers of 120 *μ*m. These films are available as sheets of 8 × 10 inches in order to allow the use when large radiation fields are needed. However, if the determination of dose in small areas is needed, it is possible to cut the films in smaller pieces without losing their characteristics. For very high-dose applications, such as industrial application, radiation processing, radiation hardness quality assurance tests, beam diagnostics and high energy physics (HEP) application, other kinds of films are available. Gafchromic HDV2 films are sensitive in the dose range from 10 to 1000 Gy. Beyond the kGys, thin foils films (active layer 12 *μ*m and polyester substrate 97 *μ*m) are used. GEX Corporation trades the B3 films (active layer: 18 *μ*m), sensitive in the range 1–150 kGy; the Far West Technology makes available FWT60 films (active layer: 18 *μ*m) for application in the range 0.5–200 kGy^35^. It is notable that the employment of RCFs as dosimeter in HEP field is a topic under study. Owing to their characteristics, RCFs can be a perfect tool for dosimetry monitoring of such a harsh environment like that of HEP facilities: no electronics typical of other detectors and therefore no interaction with the electric and magnetic fields. However, a mixed radiation field of such facilities is very complicated to study with RCFs, being composed of different radiation types and energies.

### Radiochromic films reading methods

To measure the dose, a physical quantity that represent the darkening of the film has to be related to the radiation dose. The relationship between this physical quantity and the dose of a set of films exposed to different doses of a known radiation field is the calibration. In the following, the principles of the commercial RCF reading techniques (scanners and densitometers) are explained, by showing an example of calibration of a set of films with an EPSON V800 Scanner. Therefore, the innovative RCF reading instrumentation is presented.

### Commercial techniques

**Figure 2:**
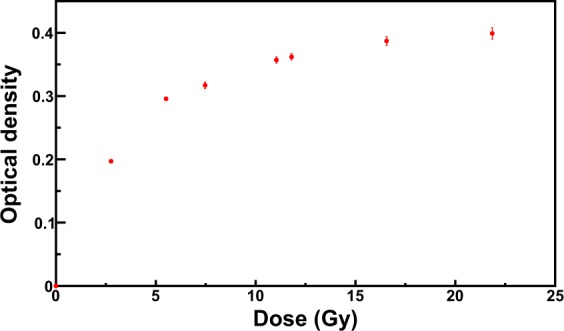
Calibration of EBT3 Gafchromic films exposed to 1 MeV electron beam. The analysis was performed with the EPSON V800 Scanner.

The physical quantity that represent the film darkness can be the Optical Density (OD) or any OD-based-functions such as netOD^1,8^. Densitometers usually provide the OD calibrated with respect to certified step-tablets. The relationship between the physical quantity representing the darkening of the films and the dose is generally non-linear; however the calibration data fit quite well to polynomial functions. The calibration performed with densitometers is therefore the OD as a function of the dose. The reading of RCFs with commercial scanners is usually performed by digitizing the image of the film. The Pixel Value (PV) can be evaluated with suited software of analysis of images and the calibration can be carried out as PV as a function of the dose or, often, as OD as a function of the dose. The OD is calculated from the PV^12,36^. As an example, Fig. 2 shows the calibration of a set of EBT3 Gafchromic films exposed to 1 MeV electron beam from ILU-6 LINAC accelerator of Institute of Nuclear Chemistry and Technology at Warsaw (Poland). The optical density increases as the dose increases. In particular, the growth is sharp up to about 10 Gy, where the sensitivity is very high. For higher doses, the optical density slowly increases until a saturation condition is reached.

### The innovative set-up

**Figure 3:**
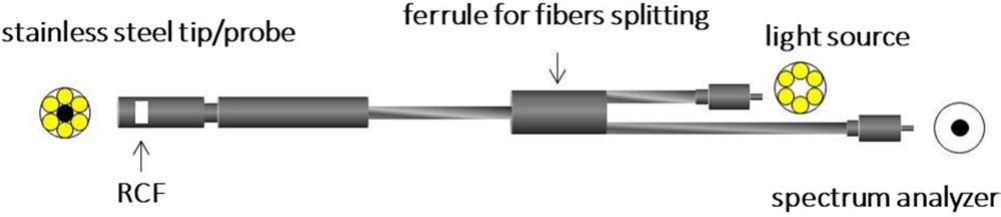
Experimental apparatus for the real-time reading of RCFs.

**Figure 4:**
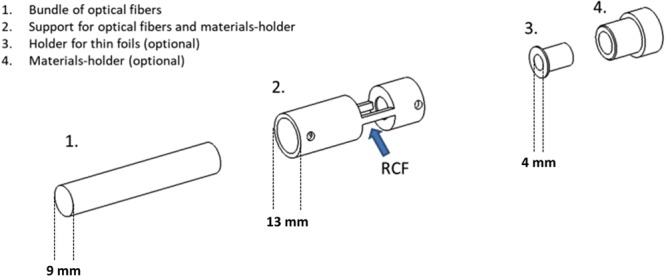
Design of mechanical elements for the support of the backscattering material.

**Figure 5:**
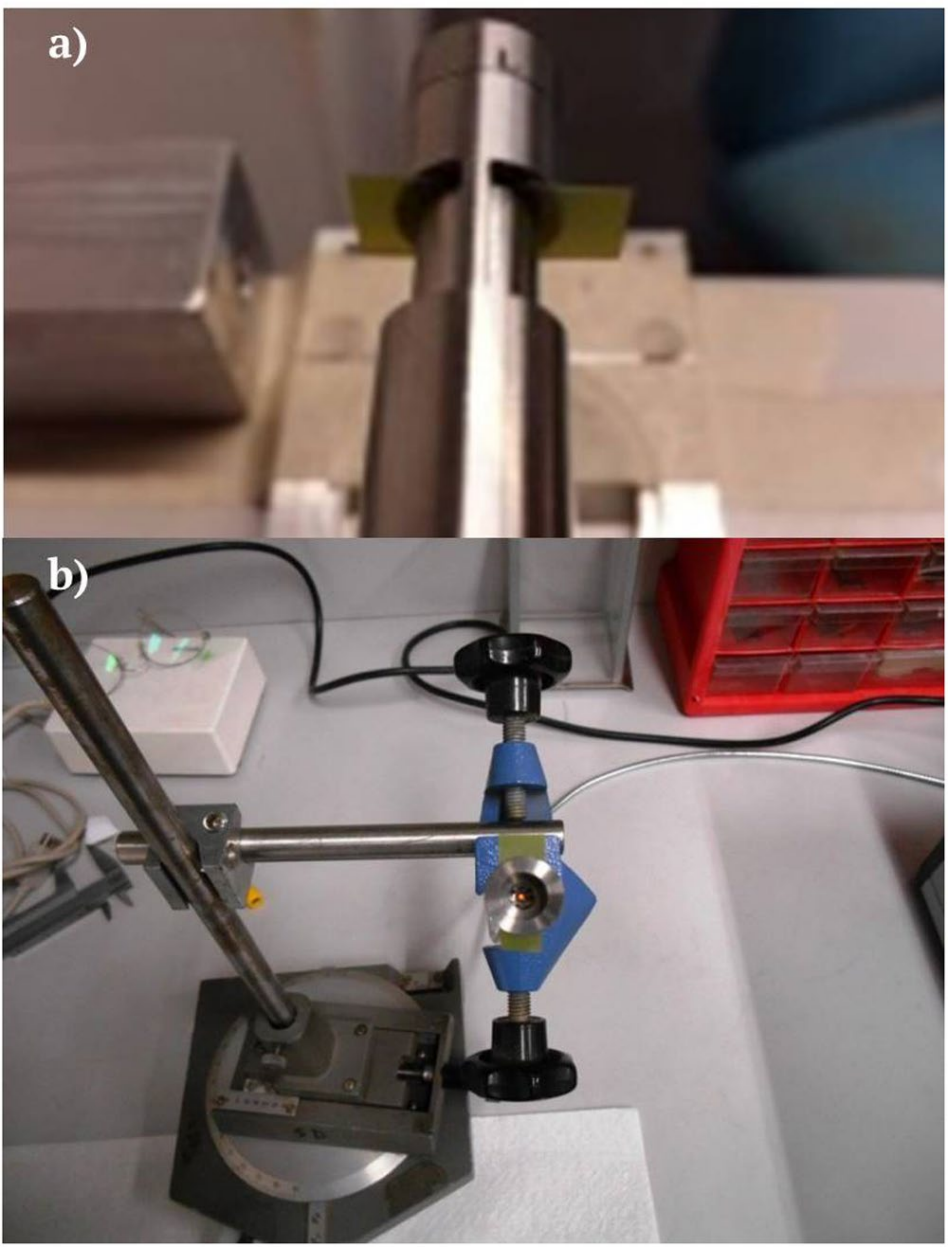
Picture of the terminating part of the optical fiber bundle with the RCF (**a**) and top view (**b**).

Figure 3 shows the schematics of the experimental apparatus employed for the real-time reading of RCFs. It basically consists of an optical fiber bundle with six illuminating fibers positioned on the sides of the bundle and one light-collecting fiber in the centre, a RCF, a light source and a spectrum analyser connected to a computer for data analysis. The six illuminating fibers transport the light from the source to the tip of the probe where the RCF is positioned. After passing into the RCF, the light is first backscattered by an appropriate material, then collected by the central fiber and therefore sent back to the spectrometer. Changes of optical properties of the film due to the darkening induced by ionizing radiation can be in this way detected in real-time. The choice of the backscattering material affects the integration-time of the spectrometer. This parameter is fundamental, being related to the sensitivity of the method: it determinates the minimum amount of time in which the spectrometer can detect sensitive changes of the light. Figure 4 shows the schematics of the elements of the terminating part of the innovative set-up. This part was designed and carried out with a twofold aim: to ensure the interaction of ionizing radiation with the RCF and to allow the backscattering of the light with enough intensity to be detected by the spectrometer. With reference to the nomenclature of Fig. 4, the element “2” is both a support for the optical fibers bundle and a holder for backscattering materials. These materials can be plastics such as ABS or Teflon, in the form of cylinders (height 1 mm and diameter 6 mm) or thin foils of Mylar (of thickness 1.5 *μ*m). It has to be noticed that the designed material-holder allows the use of the RCF itself as backscattering material, if needed. This is very useful if the RCF has an opaque layer; this is the case, e.g. of Gafchromic XR- models. The element “4” allows the hosting of the plastic cylinder. This can be screwed and fixed on the element “2”. If the thin foils are used, the element “3” can be inserted in the material-holder “4”. The elements 2-3-4 are holed. For the case of thin transparent RCFs such as EBT3 Gafchromic film model, the only absorber is the plastic cylinder or the 1.5 *μ*m Mylar foil that operate as light mirror. For opaque films such as as XRQA2 Gafchromic film model, the elements 3 and 4 are omitted so that there is no absorber between the RCF and the incident radiation. The bundle for the optical fibers can be made of different materials such as stainless steel, plastics (less interacting with ionizing radiation) or, if necessary, the single fibers can be used. In this work the stainless steel probe was used. During the operation, the whole apparatus is positioned in the irradiation area. The light source used for this prototype is the “AvaLight-DH-S-BAL Balanced Power” and the spectrometer “AvaSpec-ULS2048XL”. The latter is controlled via computer. The RCF is positioned in the material holder as shown in Fig. 5(a). Figure 5(b) shows the top view of the optical fiber bundle with the Mylar foil and the red spot light. The effective area of the RCF samples involved in the dose reading depends on the size and position of the illuminating and collecting fibers within the terminating part of the probe; in particular, referring to pictures shown in Fig. 5, the effective area corresponds to that of a circle with a diameter of three fibers having a core of 200 *μ*m. Nevertheless, in order to allow a practical and reliable insertion into the readout system, RCF samples of 8 × 20 mm^2^ were employed.

## Results

The innovative dosimetry method was successfully tested with several types of RCFs. In order to demonstrate the operating principle of the method, the results, in terms of light spectra and dose calibration curves, of irradiations of EBT3 and XR-QA2 Gafchromic films with ^60^Co gamma rays will be discussed in the following. Several physical physical quantities can be exploited for the calibration of the films, depending on the dose range to be investigated. This property gives a high level of versatility to this method, allowing the extension of the dynamic range of some types of films by more than one order of magnitude.

### Experimental

**Figure 6:**
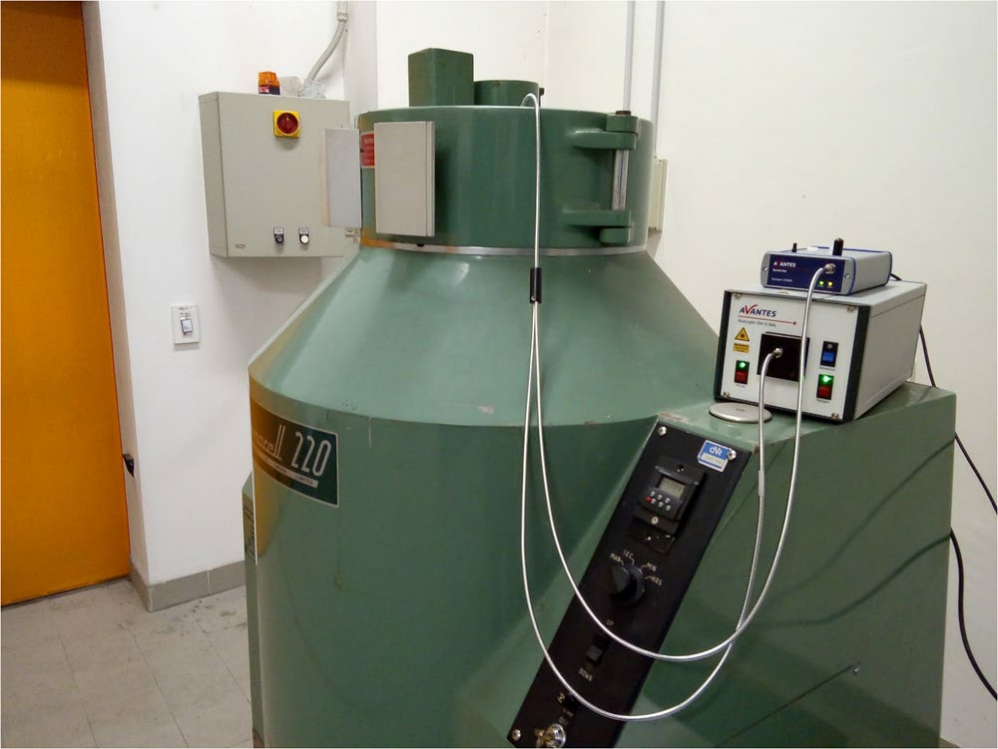
Picture of the Gammacell220 of ISOF-CNR at Bologna. During the operation the optical fiber probe is inside the irradiator. The light source and the spectrometer are remotely controlled.

EBT3 Gafchromic film samples were exposed to ^60^Co-gamma rays from ISOF-CNR gamma facility at Bologna (Italy). ISOF-CNR is equipped with two Gammacell220, providing High Dose-Rate (HDR) of 2.59 Gy/min and Low Dose-Rate (LDR) of 0.10 Gy/min from the decay of the radioactive isotope ^60^Co (half-life: 5.272 y). This undergoes *β*^−^ decay to the first and second excited states of ^60^Ni, which in turn decays via gamma emission with energies of 1.17 and 1.32 MeV. The two irradiators consist in self-shielded vertical cylinders with the centre surrounded by ^60^Co vertical rods. The dose-rates of the irradiators are determined by the activity of the ^60^Co rods. An opening in the upper part of the irradiators allows the positioning of the samples to be irradiated. During the irradiation, the fiber bundle and the film, i.e. the terminating part of the RCF reading set-up shown in Fig. 5 were positioned in the centre of the irradiator, while the light source and the spectrometer were remotely controlled. The radiation dose in the position of the RCF was previously measured with conventional RCF dosimetry. Since the radiation field of ^60^Co Gammacell is uniform and omnidirectional, the orientation of the detector for this specific irradiation is not important. In the more general case of a directional field, the new RCF reading method operates with the fiber bundle coaxial to the radiation beam (or the RCF perpendicular to the radiation beam). The film was continuously exposed to radiation and the readout performed at times fixed by the integration time. A picture of the set-up during operation is shown in Fig. 6.

**Figure 7:**
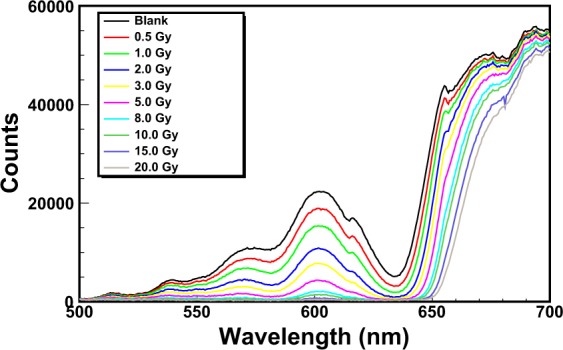
Spectra of an EBT3 Gafchromic film exposed to ^60^Co gamma-rays from the HDR-irradiator. The integration-time is set to 1.5 s.

Figure 7 shows the spectra corresponding to the exposure of an EBT3 Gafchormic film in the HDR-irradiator for ten minutes irradiation. In this first experiment the holder with the Teflon cylinder was used, implying an integration-time of the spectrometer of 1.5 s. The spectra are zoomed in the wavelength region 500–700 nm. The spectrometer acquires one spectrum for each integration-time (1.5 s). This amount of time corresponds to a dose on the film of 64 mGy. The effect of the ionizing radiation on the film consists in an integral reduction of the counts. As a consequence of this, the different curves represent the trend in time of the darkening of the film, that is the trend of the dose absorbed by the film. The first curve of Fig. 7 (blank) corresponds to 0 Gy on the film (no exposure to ionizing radiation). The second curve, with a reduced number of counts, corresponds to a dose of 0.5 Gy. The third curve (1.0 Gy) has a subsequent reduction of the counts and so on. The maximum dose achieved in this experiment was 30 Gy. The counts recorded by the spectrometer are affected by experimental uncertainties mainly related to the temperature of the light source. The contribution of the uncertainty of the counts due to the reproducibility was estimated to be less than 0.1% for a 2 hour acquisition made after 40 minutes from the switching on of the light source (the time needed for the warm-up). However, in order to take into account any source of uncertainty (e.g. possible instabilities of the light source) the overall uncertainty on the counts was considered the square root of the counts, in agreement with the *Poisson* statistics. This conservative approach overestimates the uncertainties of the counts of all calibrations reported in this work, without affecting the precision of the method. In fact, the uncertainty of the counts was evaluated to be in any case less than 1%.

### Dose calibration of EBT3 Gafchromic films

The observation of the spectra of Fig. 7 suggests that the calibration can be performed by means of any reasonable physical quantity. For example, the wavelength *λ*_*low*_ = 635 nm, corresponding to the valley of the spectra of Fig. 7, can be used for an optimal discrimination of low doses.

**Figure 8:**
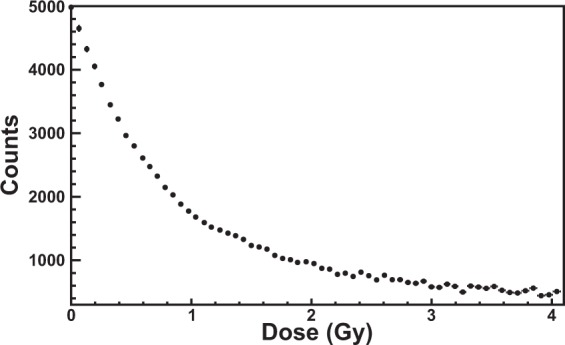
Counts corresponding to the wavelength *λ*_*low*_ = 635 nm as a function of the dose. Above 4 Gy this physical quantity is not useful for the calibration (saturation).

Figure 8 shows the counts of the curves of Fig. 7 corresponding to the wavelength *λ*_*low*_ as a function of the dose. The uncertainty of dose values (evaluated to be less then 0.2% for all the calibration plots) was obtained by the propagation of the statistical uncertainties of the integration-time and dose-rate (both less than 0.1%). The physical quantity *λ*_*low*_ is very sensitive up to about 2 Gy. In fact, with reference to Fig. 7, the gap of the counts between the first and second curve (blank and 0.5 Gy) is much bigger than that between the second and the third curve. This physical quantity saturates above 4 Gy; beyond this value the determination of dose changes is not possible.

**Figure 9:**
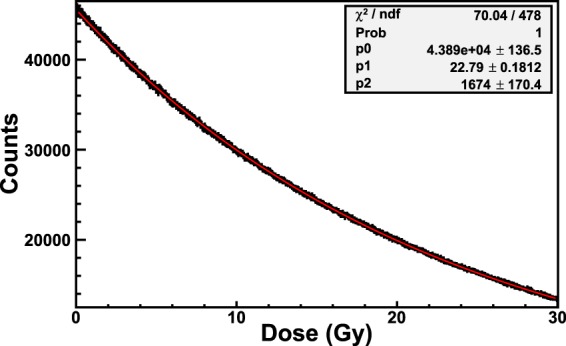
Calibration of an EBT3 Gafchromic film exposed to ^60^Co-gamma rays of the HDR-irradiator. The counts corresponding to the wavelength *λ*_*low*_ = 663 nm are used for the calibration. The red curve is the exponential function that best fits the data. The values of *χ*^2^/*ndf*, *χ*^2^-probability and the fitting parameters are shown in the inset.

In order to get a good discrimination of higher doses, the wavelength *λ*_*high*_ = 663 nm can be used for the calibration. Figure 9 shows the calibration of the film obtained by recording the counts of the curves of Fig. 7 corresponding to the wavelength *λ*_*high*_. This physical quantity allows very precise discrimination of dose in a wide dose range, from 1 Gy to more than 30 Gy. The red curve is the exponential function (Eq. 1) that best fits to the experimental data.

1 $$C={p}_{0}\cdot \exp (\,-\,D/{p}_{1})+{p}_{2}$$

Eq. 1 relates the counts (*C*) with the dose (*D*) by means of the fitting parameters *P*_0_, *P*_1_ and *P*_2_. The goodness of the fit was quantified by means of the chi-squared (*χ*^2^) test. The values of the reduced chi-square (χ^2^/*ndf*, where *ndf* is the number of degrees of freedom) and *χ*^2^-probability, respectively 0.2 and 1, of the calibration of Fig. 9 express the excellent agreement of the fitting function function with the experimental data. The inset of Fig. 9 reports the values of *χ*^2^, *χ*^2^-probability and the best estimates of the fitting parameters and of the statistical uncertainties. It is noticeable that the value of the reduced chi-square less than 1 implies an overestimation of the variances, accordingly to the conservative hypothesis of the *Poisson* statistics.

The wavelength *λ*_*low*_ is not useful for the determination of doses greater than 2 Gy, although it is a very powerful physical quantity for the discrimination of low doses. The minimum amount of dose detected by the spectrometer is determined by two factors: the dose-rate and the spectrometer integration-time (1.5 s). This value is fixed by the material employed for backscattering the light, i.e. the Teflon cylinder. With the aim of investigating a method for a better discrimination of low doses, an irradiation of an EBT3 Gafchromic film with the LDR-irradiator (dose-rate: 0.10 Gy/min) was carried out. Furthermore, in order to reduce the integration-time, the 1.5 *μ*m Mylar foil was used as backscattering material. This set-up allowed the setting of the integration-time of the spectrometer to 246 ms. In this way, the spectrometer detects changes of the darkening of the film in a smaller time with respect to that with the Teflon cylinder.

**Figure 10:**
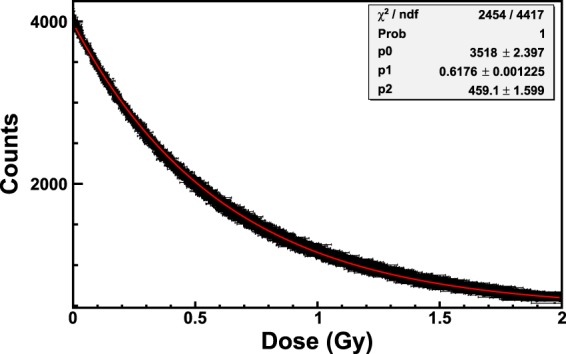
Calibration of an EBT3 Gafchromic film exposed to ^60^Co-gamma rays of the LDR-irradiator. The counts corresponding to the wavelength *λ*_*low*_ = 635 nm are used for the calibration. The red curve is the exponential function that best fits the data. The values of *χ*^2^/*ndf*, *χ*^2^, -probability and the fitting parameters are shown in the inset.

Figure 10 shows the calibration of the EBT3 Gafchromic film obtained by recording the counts of the curves of the spectra corresponding to the wavelength *λ*_*low*_ as a function of the dose. The amount of dose absorbed by the film in one integration-time is 410 *μ*Gy. The dose-trend of the experimental data of Fig. 10 is the same of Figs 8 and 9, i.e. the data are very well represented (see values of *χ*^2^/*ndf* and *χ*^2^-probability in the inset of 10) by an exponential function (red curve in Fig. 10).

### Dose calibration of XR-QA2 Gafchromic films

As already mentioned in the previous sections, some types of RCFs have a particular structure which is suitable for this innovative method. For example, XR-QA2 Gafchromic films have an opaque layer, allowing the use of the film itself as backscattering material. For this reason, an irradiation of an XR-QA2 Gafchromic film in the LDR-irradiator was carried out.

**Figure 11:**
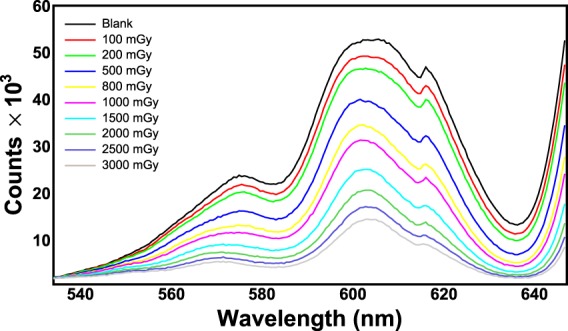
Spectra of an XR-QA2 Gafchromic film exposed to ^60^Co gamma-rays from the LDR-irradiator. The integration-time is set to 630 ms.

Figure 11 shows the spectra corresponding to the exposure of an XR-QA2 Gafchormic film in the LDR-irradiator for ten minutes irradiation. The spectra are zoomed in the wavelength region 530–640 nm.

**Figure 12:**
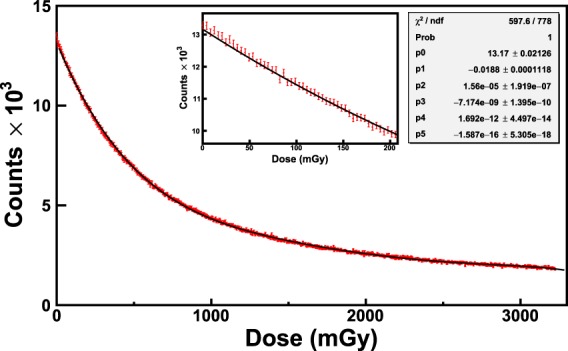
Calibration of an XR-QA2 Gafchromic film exposed to ^60^Co-gamma rays of the LDR-irradiator. The counts corresponding to the wavelength *λ*_*low*_ = 635 nm are used for the calibration. The black curve is the exponential function that best fits the data. The zoom of the main plot in the range of dose [0–200 mGy] is shown in the first inset. The values of *χ*^2^/*ndf*, *χ*^2^, -probability and the best estimates of the fitting parameters are shown in the second inset.

The integration-time was set to 630 ms. The dose absorbed by the film in one integration-time is therefore 1 mGy. Figure 12 shows the calibration of the XR-QA2 Gafchromic film obtained by recording the counts of the curves of Fig. 11 corresponding to the wavelength *λ*_*low*_. It is noticeable that the Gafchromic film XR-QA2 shows characteristic spectra and calibration curves similar to that of the EBT3 model. In particular, the fitting curve is the sum of two exponential functions of the type of Eq. 1. The values of the fitting parameters are shown in the inset of Fig. 12. The values of *χ*^2^/*ndf* and *χ*^2^-probability are 0.8 and 1 respectively.

### Validation of the new RCF real-time reader

**Figure 13:**
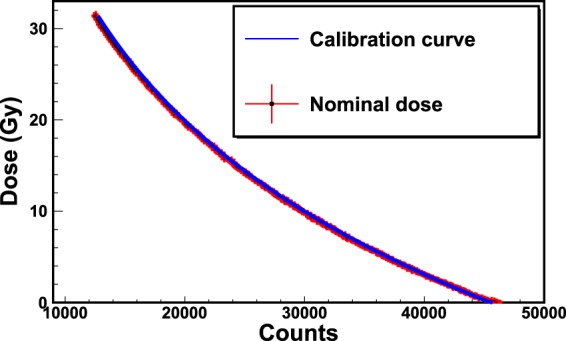
Validation of the new RCF real-time reader. The plot shows the dose as a function of counts for an exposure of an EBT3 Gafchromic film to the HDR irradiation. The nominal doses (black dots) are compared to the calibrated doses (blue curve). The maximum uncertainty was estimated by computing the percentage difference (Δ %) between calibrated and nominal doses. Figure 14 shows the maximum uncertainty as a function of dose for the values of Fig. 13.

In order to validate the obtained calibration curves, additional films (from the same batch) were exposed to known doses. The doses obtained with the calibration curves of Figs 9–12 were compared to the nominal doses, allowing the evaluation of the corresponding uncertainties. Figure 13 shows the output plot of the new reader (Dose on vertical axis; Counts on the horizontal axis) for an exposure of an EBT3 Gafchromic film to the HDR irradiatior. The doses (blue curve) were computed by reversing the calibration curve of Eq. 1 and compared to the nominal doses (black dots).

The maximum uncertainty is within 2% from 2.5 Gy on. Similar procedures were carried out for the data of Figs 10 and 12 leading to maxima uncertainties within 4% from 100 mGy and within 8% from 30 mGy respectively. These uncertainty values can be reduced by optimizing the integration-time of the spectrometer to the wavelengths of interest for the calibration. Moreover, the integration-time can be further reduced by using thin high-reflecting materials. These procedures provide also the value of the minimum detectable dose obtained with this method. This point will be object of next studies.

## Discussion

In this work an innovative method, based on opto-electronic instrumentation, is proposed for the real-time reading of RCFs. The method is based on the use of optical fibers that transport the light from a source, such as a halogen or deuterium light source, up to the RCF. During the exposure to ionizing radiation, the optical changes of the film are detected in real-time by a light-collecting fiber and sent back to a spectrometer for data analysis. A proper scattering material can be used, if needed, to increase the amount of light in the collecting fiber. This allows the reduction of the integration-time of the spectrometer, affecting ultimately the minimum detectable dose.

The method was tested for several RCF types, showing excellent performances in terms of accuracy and precision. The results of the irradiations of two types of films (EBT3 and XR-QA2 Gafchromic model) to ^60^Co-gamma rays at two dose-rates (2.59 and 0.10 Gy/min) are reported in this work. The versatility of the method relies on many factors, among which the capability of performing the dose calibration by means of more criteria of data analysis. For example, the counts corresponding to the characteristic valley of the spectra, i.e. the wavelength *λ*_*low*_ = 635 nm, can be put in relation to the dose on the RCF. The subsequent calibration, based on a proper choice of the backscattering material, shows that the dynamic range of some types of films is higher with respect to what declared by the manufacturer. In fact, EBT3 Gafchromic films, intensively employed in medical physics for dosimetry quality checks in radiation therapy, are sensitive up to dozens of Gy with the analysis with commercial reading instruments. The proposed dosimetry method clearly allows the detection of doses of hundreds of mGy with EBT3 Gafchromic films with precision of 4%. These uncertainties can be further reduced for lower doses by optimizing the integration-time and by using high-reflecting back-scattering materials. Different data analysis techniques can be exploited for the investigation of a specific dose range. For example, by recording the counts of the spectra corresponding to the wavelength *λ*_*high*_ = 663 nm, it is possible to perform a calibration for better discriminate the high doses. It should be noted that the spectra shown in Figs. 7 and 11 can be converted in principle into optical density (OD) spectra, provided that they are normalized to the first acquired spectrum corresponding to a zero dose. By way of example, Fig. 15 shows the spectra of Fig. 7 up to 3 Gy converted into OD. The OD was calculated according to the following expression:

$$OD(\lambda )={\mathrm{log}}_{10}(\frac{{I}_{0}(\lambda )}{I(\lambda )})$$

where *I*_0_(*λ*) is the light intensity measured before the exposure and *I*(*λ*) is that measured during the exposure to radiation. The OD spectra exhibit two main peaks respectively at 635 nm and 585 nm, revealing high similarity to those shown in the literature^37^ as proof of the consistence of the proposed method with standard light-transmission-based approaches. The evaluation of measurement uncertainties has been made by means of a precautionary approach, based on the *Poisson* statistics, allowing uncertainties within 1% for all the calibration plots (Figs 9–11).

**Figure 14:**
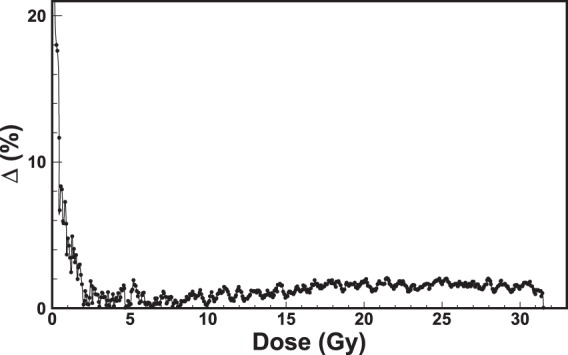
Percentage difference (Δ %) as a function of dose for the values of Fig. 13.

**Figure 15:**
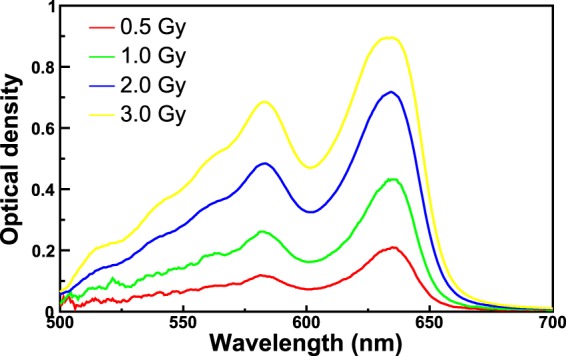
Optical density spectra obtained by evaluating the OD of the spectra of Fig. 7.

The advantages of the proposed method with respect to the state of the art are manifold. Firstly, the real-time dosimetry with RCFs can be performed by monitoring the dose from a remote position with an optical fiber probe, exploiting the potential of optical fibers such as micrometric thickness, low attenuation coefficient and no interaction with electromagnetic fields. The irradiation of small areas (less than 1 mm^2^) of commercial RCF allows multiple exposures and readings of the same RCF up to the saturation. As a result a National Patent was filed in January 2018. RCF dosimetry finds applications in any field of the radiation physics, each one with a specific target. Current author’s research efforts are devoted to the study of the miniaturization of the present probe, in order to make it competitive with other dosimeters and detectors in some specific application field, such as the dosimetry of the irradiations of the electronic devices for space and terrestrial radiation hardness applications. Finally, the miniaturization of the present prototype up to sub-millimetre dimensions can pave the way to interesting applications of the proposed method such as *in-vivo* dosimetry.

## Data Availability

The data analysed during the current study are available from the corresponding author on reasonable request.
